# Role of change leadership in attaining sustainable growth and curbing poverty: A case of Pakistan tourism industry

**DOI:** 10.3389/fpsyg.2022.934572

**Published:** 2022-11-02

**Authors:** Fatima Bashir, Zara Tahir, Amna Aslam

**Affiliations:** ^1^Department of Business Studies, Air University School of Management, Air University, Islamabad, Pakistan; ^2^Faculty of Management Sciences, International Islamic University, Islamabad, Pakistan

**Keywords:** sustainability, change leader, sustainable tourism attitude, resilience, sustainable organizational performance

## Abstract

This study has proposed to apply change leadership as a vehicle forward for sustaining the growth of the tourism industry to eradicate poverty through the Pakistani tourism industry. Applying a mixed method approach, this article has attempted to uncover the role a change leader can play to help achieve the United Nations’ sustainable development goals of poverty reduction. In this study, one of the authors interviewed stakeholders of the tourism industry to find out the major drivers of the industry and identify the leadership style that may prove to be effective in the said industry. In the next phase of the study, a survey method approach was used where different tourism companies and hotel chains were included for analysis. The study aimed to check employee’s perception of change leadership and its impact on their resilience and ultimately the sustainable organization performance of companies’ operationalizing in the sector with moderating impact of sustainable tourism attitude in times of a crisis. In total, 430 full-time workers in the tourism industry were contacted for a self-administered survey achieving a response rate of 83%. The findings of the study confirmed that in the presence of a change leader, the process of adaptation to sudden changing situations amidst financial crises, pandemic, and climatic change, becomes bearable and employees can cope with the work situations without pushing them to quit the industry. The study has a significant contribution toward a rapidly growing and focused industry, which can play a major role in the economy of any country leading toward job creation and poverty reduction.

## Introduction

The interest of researchers and competitiveness in tourism sustainability has significantly enhanced radically owing to major changes that took place in supply and demand in the tourism industry led by globalization (Santos et al., 2022). Along with competitiveness, the ever-changing world, and shifting focuses of social values, recognition of how past growth has negatively affected the environment has also led to attention toward sustainability more than ever before. Highly visible issues such as global warming, shrinking water bodies, and loss of biodiversity call for change in the practices of the human race to sustain the environment before it is too late (UNWTO, 2020). Change has been the only constant in the quests for sustainable tourism presenting the industry with economic, environmental, as well as social issues that require substantial changes in traditions and attitudes of stakeholders of the said industry (Kernel, 2005). Issues such as severe climatic changes, poverty, resource shortages, unstable politics, and globalization have been able to attract researchers as well as practitioners in light of the United Nation’s Sustainable development goals (Caffaro et al., 2019). The current study in this context serves as a source to open policy making and further investigations to explore the tourism industry to curb issues such as climatic changes and poverty in a progressing economy such as Pakistan.

The employees in the tourism industry have been facing a lot of stress during the pandemic as the overall scenario of the world drastically changed. With frequent lockdowns and travel bans, the industry suffered massive losses, and it became a challenge for employees not to turn over and change their course of action (Gössling et al., 2021). World Travel and Tourism Council (WTTC) declared an estimation in September 2020, indicating that 121 million jobs in the tourism industry creating the worst social and economic crises calling for research in the domain to identify leadership styles and employee attitudes, which can contribute to the sustainable performance of organizations’ operations in the tourism industry.

Despite that, the industry has been surviving if not thriving in the scenario. If provided with an effective leadership style in such a rapidly changing scenario aiding the development of effective attitudes of employees, this can be a potential source to reduce poverty through job creation and sustainability (Anwar, 2017). Literature shows while such times of crises require changed policy and tactics, as change management is required, the role of effective leadership cannot be ignored in introducing and sustaining change (Gill, 2002). This research study is based on mixed methodology where in the study one a qualitative analysis has been conducted identifying the most effective leadership study in the scenario and the desired employee attitudes which may contribute toward a sustainable tourism industry. The next phase of the study tests the identified themes in a structured model conjugated with theory in a quantitative analysis leading to the inference of results for policy making and recommendations.

## Literature review

### Change leadership and employee resilience

In today’s highly dynamic world, the hardcore challenge for organizations in the 21st Century highly depends on the capability to adopt change innovatively to gain a competitive advantage (Wijayati et al., 2022). However, innovative leaders who adopt change quickly play an important role to advance sustainably in this competitive environment (Kistyanto et al., 2021). Leadership is the process of mutual interaction among the leaders and their subordinates. However, the role of a leader is to shape the behavior of their employees in a way that leads toward the accomplishment of the organization’s goals. Moreover, the role of leadership can be seen from multiple aspects, such as “traits, behaviors, influence, interaction, role relationships, and occupation of an administrative position” (Yukl et al., 2013). It further can be also described as the leaders themselves and their group of people who shared firm mutual interests (Ali and Shastri, 2010). In past research studies, scholars have identified various leadership styles which are practiced in organizations based on the extent of need. However, in today’s fast-growing and competitive world, the sustainability in the tourism industry has knowingly enriched drastically owing to major changes that took place in supply and demand in tourism industry lead by globalization (Santos et al., 2022). In addition, in a previous research study, it was found that managing change in an organization has become a major issue and challenge for a leader to effectively deploy it (Herold et al., 2008). In the current times change leadership is proving to be a more adjustable style of leadership, which can increase the productivity of the organizations and employees in the changing circumstances of organizations.

Moreover, in the context of change leadership, which is defined as “the style of a leader to make changes with the desire and vision for the future for organizations that need change; the desire and ability to carry out and direct change, as well as the willingness and ability to accompany a change process, to produce better organizational conditions (Amir and Mangundjaya, 2021).” In addition, change leadership is also known as the implementation of the whole change process successfully (Liu, 2010). In addition, along with the strategically developed long-term vision and goals, however, to attain those goals, the mobilization of the energy among the members of an organization to attain the mutual shared goal (Hooper et al., 2010). In a previous research study, the basic difference between the “change management” and “change leadership” is holds a major difference. The change management, refers as the “set of essential tools or structures intended to keep any change effort under control. The goal is often to minimize the distractions and impacts of the change.” Whereas the change leadership “concerns the driving forces, visions, and processes of large-scale fuel transformation (Gupta, 2011).” Change leadership is a combination of the desired traits of a leader that are supportive of the employees of the organization but also strategically in line with the organization’s vision and mission.

In addition, the leaders are fully accountable for the efficient and effective implementation of the change process in an organization and for encouraging and motivating employees in a manner that develops the change culture successfully in an organization (Madsen et al., 2005; Herold et al., 2008). Moreover, for the sustainability of organizations, there is a massive need to provide awareness and urgency among the employees for the need to adopt to change plus to develop the sense of belonging in the process of change in an organization (Sundström and Zika-Viktorsson, 2009). However, an individual readiness to change is a “person’s beliefs, behavior, and intentions toward the required change and is related to their perception of the individual and organizational capacity to achieve success in the change (Armenakis et al., 1993).” Moreover, the concept of employee resilience is in which “people are more capable to face unprecedented changes and adapt effectively to challenging roles, tasks, and situations (Shin et al., 2012).” Change is the only constant around the world and especially in times of unprecedented changes, there is a requirement of unprecedented measures of the leader to help them adjust through the tough times of change coming out of their *status quo*.

The construct of employee resilience was developed based on the conceptualization of organizational resilience (Näswall et al., 2013), which is defined as “a function of an organization’s overall situation awareness, management of keystone vulnerabilities, and adaptive capacity in a complex, dynamic, and interconnected environment” (McManus et al., 2008). However, the concept of employee resilience is further explained in which “people are more capable to face unprecedented changes and adapt effectively to challenging roles, tasks, and situations (Shin et al., 2012).” However, when the organization helps and supports its employees for the development and boosting during the change process and challenging times or circumstances that help them to adapt to the change successfully by boosting the capability of their employees in an effective manner.

In addition, employee resilience includes difficulty and complexity at the start but they result as positive adoption of change. When the embracing of change influences employee resilience with the resource-utilizing and adaptive capacity, it leads toward developing of organizational resources (De Rossi, 2013). In past, it was found that change leadership can influence highly resilient individuals which have better coping and adaptive abilities when faced with adversity, such as work-related stress. In addition, it is found that when employees are persistently motivated and augmenting to find innovative methods and bumped into challenges by coping with the change, they are likely to cultivate better change readiness, which encourages employee resilience (Sundblad et al., 2013). Therefore we hypothesize:

**H1: Change leadership has a positive and significant effect on employee resilience.**

### Employee resilience and sustainable organizational performance

In today’s fast-growing and competitive world, business paradigms are shifting due to the increase in innovations, the revolution of digital technologies, and sustainability is the new emerging contemplation in relation to human and organizational practices in every aspect. Moreover, in organizations, the vital foundation in achieving a competitive advantage to survive and compete is sustainability, which brings value to shifting the economic paradigm (Pieroni et al., 2019; Ferasso et al., 2020). However, sustainability is a new conception, which has gained grasping attention and consideration of academicians, business leaders, practitioners, policy makers, and requires further expansion of the business literature (Ferreira et al., 2021). In addition, in the past, sustainability-related issues in business were discovered around the world, and it becomes an emerging issue (De Massis et al., 2021). In addition, the sustainability-related issues have mostly focused on the consumer behavior or products and services-related perspectives but gained less focus and attention on the employee-related perspective to maintain sustainable organizational behavior (Charbonnier-Voirin et al., 2010). Organizational sustainability performance is defined as “a dynamic process that necessitates achieving short-term performance (meeting current needs) without compromising long-term performance (meeting future needs) of the triple bottom lines—financial, social, and environmental (Eccles et al., 2014).” However, the employees in the tourism industry have been facing a lot of stress during the pandemic as the overall scenario of the world drastically changed, in this kind of situation, efficient organizations take calculated moves by keeping their business sustainable and protecting themselves from huge downfall since most of the organizations endeavor for the organizational sustainability (Merriman et al., 2016). To cope with the uncertainty in the industry and unstable economy as much as the role of leadership is critical so is the internal ability of the employees to fight adversities with their internal power to resist any collapse and to keep it going is also critical.

The importance of developing resilience in an organization is to be protective toward employees to prevent the contrary situations and adverse effects in the complex work situations (Howard, 2008). Furthermore, in previous literature, it was found that these practices followed in an organization, such as meaningfulness, competencies, and autonomy, can provide a positive and healthy work environment and improve work experience (Fredrickson et al., 2003). Resilience is an aptitude to “anticipate, prepare, respond, and adapt” to events. The organizations in which employee resilience exists, they are more adaptive, positive, and manage “rapid changes in environmental, social, technological, and economic systems” (Chuang and Huang, 2018). There have been many studies in the past that have shed light on the importance of resilience in multiple factors of organizational life but when it comes to sustainability the role of resilience in maintaining it cannot be ignored.

Additionally, the undeviating association between sustainability and resilience has gained the researcher’s attention in different aspects, such as the application of research on water (Santora and Wilson, 2008), community resilience (Berkes and Ross, 2016), and urban resilience (Meerow and Newell, 2019). Resilience also shares the same aspect, such as sustainability, and thus calls for more research separately (Marchese et al., 2018). The research suggests that during challenging and crisis times, an organization where their employee’s resilience is high is the best place to work (Carvalho and Areal, 2016). When the organization helps and supports its employees for the development and boosting during the change process and challenging times or the circumstances that help them to adapt to the change successfully by boosting the capability of their employees effectively, and when the employees recognize that the organization is valuing their contribution and providing them the required help, they reciprocate with efforts to increase the organizational performance (Imran and Aldaas, 2020). Employee resilience directly shapes the performance of an organization and can increase and contribute positively to organizational performance (Stajkovic et al., 2006; Luthans and Youssef, 2007; Ollier-Malaterre, 2010; Robertson et al., 2011; Blumberg et al., 2014; Scarbrough et al., 2015).

However, numerous organizations perceive employee resilience as an asset and quality trait for an organization to enhance its performance. Execution of resilience needs an effective leader to overcome the crisis, which is oriented toward superior performance and focus on change (Malmi and Brown, 2008). Moreover, an organizational culture by proactively implementing change leadership can create employee resilience, which leads toward the sustainability, enhancement, and success of the organization (Alesi, 2008). The finding reveals that organizational resilience is positively and significantly associated with organizational performance (Malik and Garg, 2017). Change leadership and employee resilience in the organization play an important role in organizational performance to meet all the challenges and issues that arise during tough situations. Therefore, we hypothesize:

**H2: Employee resilience positively relates to a sustainable organizational performance.**

### Sustainable tourism attitude, employee resilience, and sustainable organizational performance

In recent times, the tourism sector has become the fastest developing and growing sector around the globe. However, in the development of the economy, it plays a major role (Dibra and Baraku, 2019). Sustainability is a new conception, which has gained attention and consideration of academicians, business leaders, practitioners, policy makers, and requires further expansion of the business literature (Ferreira et al., 2021). In addition, in the past, sustainability-related issues in business have been discovered around the world, and it becomes an emerging issue (De Massis et al., 2021). The conceptualization of sustainable tourism was first noticed and discussed by Butler (1999) and then onward gradually developed and gained the attention of the United Nations in the sustainable development agenda (United Nations, 2018). Moreover, it is found important in terms of extending the body of knowledge in sustainable tourism as well as understanding its practical implementation (Hsu et al., 2020). However, in the literature, it is found that the research on sustainable tourism is scare and does not provide enriched understanding, clear target, and its practicality; therefore, obstructing the conversion of traditional tourism into sustainable tourism (Bramwell and Lane, 1993; Wheeller, 1993; Harrison, 1996; Stabler and Goodall, 1997; Butler, 1999).

Moreover, in organizations, the vital foundation in achieving a competitive advantage to survive and compete is sustainability, which brings value to shifting the economic paradigm (Pieroni et al., 2019; Ferasso et al., 2020). Highly visible issues such as global warming, shrinking water bodies, and loss of biodiversity call for change in the practices of human race to sustain the environment before it is too late (UNWTO, 2020). Change has been the only constant in the quest for sustainable tourism presenting the industry with economic, environmental, as well as social issues that require substantial changes in traditions and attitudes of stakeholders of the said industry (Kernel, 2005). Meanwhile, during the COVID-19 pandemic for shaping of the attitude of tourism industry stakeholders, various scholars conducted research to balance the tourism economy and employees’ attitude toward sustainable tourism (Pichlak and Szromek, 2021). Furthermore, to develop sustainable tourism attitude among the employees and communities, it is important to provide utmost support, such as accessible resources, to develop an attitude toward sustainability (Jones, 2019). However, it is important to find out to what extent the sustainable tourism attitude can influence the relationship between employee resilience and sustainable organizational performance. Therefore, we hypothesize:

**H3: Sustainable Tourism Attitude can moderate the relationship between employee resilience and sustainable organizational performance.**

## Methodology

**FIGURE 1:**
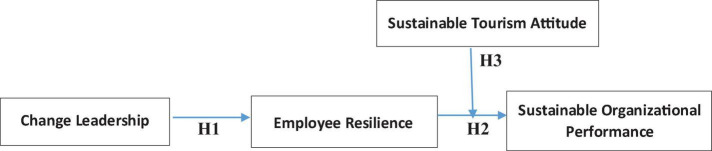
Theoretical framework.

In this research we are following a mixed method research in order to assess the both qualitative and quantitative side. However, the study 1vis based on qualitative data which is based on interviews and study 2 is based on survey data based on theoretical framework as demonstrated in Figure 1.

### Study 1 – Qualitative study

We conducted 21 qualitative, in-depth interviews with participants from across six cohorts (*N* = 231, 13%) who had been working in the tourism industry in Pakistan over the past 5 years. The participants were selected using convenience sampling based on a referral to ensure that various experiences and views were collected. The interviews were conducted in the initial phases of the study with a mean duration of 20–25 min (depending on how long respondents took to share their experiences) using both face-to-face and skype interface by the authors, the interviews were recorded and were transcribed verbatim. The schedule of the interviews was developed by authors using foundations of literature and the following themes were included to explore the perceptions and thinking of the participants:

• The benefits of working in tourism industry
• Major challenges faced by the tourism industry intimes of the pandemic
• Challenges faced in practice by the sector and how it can contribute to poverty alleviation and conservation of climate. How do they think the industry can grow?
• Find out the major drivers of the industry and what kept employees running in times of crises?
• Identify the role of leadership that may prove to be effective in the said industry in times of crises to promote sustainability in the industry.
• How their attitudes got affected in times of crises?

To improve the validity of the data collected and to curb any kinds of biases and social desirability, several systematic steps were taken. The interviewers tried their best to avoid any leading and loaded statements. Open discussions were conducted, and interviewees were allowed to share their voices to improve the industry in which they were working by sharing their personal experiences with their managers. As there are no published guidelines to conduct an unstructured interview, the researchers followed the conventions and implemented the steps used in most studies as recommended by past researchers for the conduct and planning of such interviews (Minichiello et al., 1990; Punch, 1998; Heyl et al., 2001; Fontana and Frey, 2005).

#### Data analysis

Content analysis (directed) was conducted to analyze the data collected in the interviews, which resulted in pre-determined themes to investigate the problem area on hand (Hsieh and Shannon, 2005). The interviews were audiotaped with the prior consent of the interviewees. The directed data were transcribed using a structured process where key concepts were used as coding categories initially. The area of interest in this study was to learn how an effective leadership style may strengthen the workforce making them more resilient in times of crises *via* an in-depth analysis of the tourism industry and elaboration of employees working in the tourism industry for the last 5 years. The pre-determined themes, in this case, were (i) effective leadership styles, (ii) employee personality traits for survival in times of crises, (iii) factors contributing to sustainable tourism industry, (iv) promising outlook of the organization’s operation in the tourism industry.

The transcripts of the interviews were carefully read and analyzed by local language–speaking authors. In the second phase of read-through, the authors in their independent analysis focused on the four theoretical themes that were pre-determined for inductive reasoning and ensuring trustworthiness, validity, and reliability of the data for qualitative analysis (Berends and Johnston, 2005; Church et al., 2019). Authors independently coded interviews and in the discussion session addressed the discrepancies observed in their analysis mutually. An exhaustive analysis of the data was done to ensure if there is any data present in the interviews which cannot be coded in the pre-determined themes and requires any subcategories, but it was found that all data fit in the categories completing the stage of data analysis. Since mixed language was used during the interviews after agreement on final codes, the themes were purely translated into English by language experts and were agreed upon by all the authors.

#### Findings

Of the 30 invited workers in the tourism industry, 11 decided on telephonic interviews for participation and another 10 participated in face-to-face interviews. Participate in telephonic interviews. The characteristics of interviewed individuals are as below in Table 1. Participants were identified as employees in tourism ET-1–ET-21 (Employees in Tourism).

**TABLE 1:** Characteristics of the participating employees from the tourism industry.

Participants	Formal leadership education	Years of experience	Impacted by pandemic
ET-1	Yes	> 5	Directly
ET-2	Yes	3–5	Indirectly
ET-3	Yes	> 5	Directly
ET-4	Yes	0–2	Directly
ET-5	No	3–5	Directly
ET-6	Yes	3–5	Directly
ET-7	Yes	> 5	Directly
ET-8	Yes	3–5	Indirectly
ET-9	Yes	> 5	Indirectly
ET-10	No	> 5	Directly
ET-11	No	3–5	Directly
ET-12	Yes	2–5	Directly
ET-13	No	1	Directly
ET-14	Yes	2–5	Directly
ET-15	Yes	3–5	Directly
ET-16	Yes	< 5	Directly
ET-17	No	3–5	Directly
ET-18	No	2–5	Indirectly
ET-19	Yes	> 5	Directly
ET-20	Yes	> 5	Directly
ET-21	No	> 5	Directly

The majority of participants were men with a few exceptions of women. Mean age of 48 years (range 25–60).

The main findings of the study revealed that the employees working in the tourism industry have been facing problems of rapidly changing work environment owing to changing policies and uncertainties about their income and working conditions. They believe that in the presence of a leader who can lead them through change would enable them to develop the capability to fight such adversities leading to the development of the sustainable performance of their organization. The findings are presented as four themes (Table 2): Leadership beliefs and values; employee personal traits; employee attitudes; Organizational growth and development.

**TABLE 2:** Main themes of the study findings.

Theme 1	Theme 2	Theme 3	Theme 4
Leadership beliefs and values	Employee personal traits	Employee attitudes	Sustainable performance
Forward and strategic thinking	Adaptability	Positive attitude	Financial performance
Decisiveness and confidence	Ability to avoid burnouts	Perceived benefits	Social impact
Communication	Strong and quality relationships	Tourists satisfaction	Environmental impact
Inspiration and enthusiasm	Dependability in crises		

#### Discussion

The data collected in the study have highlighted several challenges that employees working in the tourism industry have faced during times of crises such as COVID-19 and how it has changed the overall outlook of work at their end. Lessons learned in the situation keeping in view how sustainability of the industry is highly critical for job sustainability of the masses and conservation of climate along with the promotion of tourism for the mental health of tourists and locals, the role of an effective leader cannot be ignored. The manager plays a vital role in creating common awareness, creating transparency, bringing in required changes, and ensuring implementation and sustainability of the results of changed policies. The discussions with all the interviewees indicated that the role of a change leader to be the most viable and effective style of leadership in such crisis situations and rapidly evolving markets.

The interviews with the employees in the tourism industry also revealed how the internal power of the employees during times of crises plays an important role in how they survive the crises and contribute toward the attainment of goals set by a change manager. No matter how much the manager persuades the employees to keep it going if they are unable to instill resilience in their followers, their common target of sustainable performance of the organization would not be possible. In conclusion, resilient employees with a sustainable tourism attitude as identified from our qualitative analysis of the workers in the industry, proves to be the link between change leadership and its impact on a sustainable organization that would ultimately contribute to the performance of the industry ultimately becoming a source of poverty reduction in the said sector.

### Study 2 – Quantitative study

#### Participants and procedures

A field survey was conducted to gather time-lagged data from a single source (i.e., self-report). Respondents from selected organizations responded to a survey questionnaire on the Change Leadership at Time 1, Employee Resilience and Sustainable Tourism Attitude were tapped at Time 2, and Sustainable Organizational Performance was tapped at Time-3. An approximate gap of one and a half months was given between Time 1, Time 2, and Time 3 responses to reduce common-method bias. With the help of the time lag approach, sufficient time was given to respondents to forget about their responses given at an earlier time (c.f. Podsakoff et al., 2012).

To select an appropriate industry for our study, we opted for the tourism industry in Pakistan. This industry seems to be the backbone of Pakistan’s GDP, as the Government predicted that tourism will contribute approximately Rs 1 trillion to the Pakistani economy. This growing industry will foster revenue generation and will create employment opportunities for the young generation. There are a lot of organizations included in the bracket of the tourism industry but due to limited resources and bounded rationality, we selected 7 organizations working in the twin cities of Islamabad and Rawalpindi. With the help of convenience sampling, we were able to target a lot of respondents.

To access the selected organizations, we used our contacts who own any tourism agency or know someone who is working for a tourism company, further their references added to the list of respondents of the study. Proper paper copies were provided to respondents with an attached cover letter on the first page. The cover letter was used to convey the purpose of the study, the confidentiality of the responses, and referring the voluntary contribution of the participants. Respondents were given clarity that their personal information will only be used for matching responses gathered at multiple time intervals. The personal information will immediately be discarded after matching at three time intervals. Approximately 730 questionnaires were distributed at Time-1 out of which 550 were complete and usable, generating a response rate of 75%. At time 2, the same respondents were contacted again and a total of 390 usable responses were generated leading to an overall response rate of 48%. Finally, the last round was conducted and a total of 306 usable responses were generated with an overall response rate of 38%.

The demographics of the study showed that 54% of men and 46% of women were part of the study. The majority of the respondents belonged to the Marketing department, that is, 49.2%, while others were from the Finance department (23.3%) and Administration/HR (27.5%). The mainstream respondents hold a Master’s degree (55.4%) while others had bachelor’s degrees (44.4%). A popular stream of respondents were middle-level managers (53.1%) followed by entry-level managers (34.7%) and top managers (12.2%). Moreover, the participants had specializations in marketing (49.2%), finance (27.5%), and HRM (23.3%). The present work experience in their organization ranges from 1 to 6 years, and the total accounts for 8–33 years.

#### Measures

The questionnaires were designed in the English language as English is the primary medium of instruction in all Higher education institutes and official communication across Pakistan. Therefore, a wide population can understand, communicate, and comprehend English easily. The previous research conducted in Pakistan also designed survey forms in English (e.g., Naseer et al., 2016).

All measures were evaluated using a common five-point Likert scale starting from 1 = Strongly Disagree to 5 = Strongly Agree.

Change Leadership. The change leadership was measured from Herold et al.’s (2008) change leadership scale, which is a seven-item on five-point Likert scale. A Sample item is “Developed a clear vision for what was going to be achieved by our department.” Chronbach’s alpha showed the reliability of the scale in the present study as 0.80.

##### Employee resilience

The employee resilience scale was developed by Näswall et al. (2015), which has nine items. A sample item is “He/she effectively collaborates with others to handle unexpected challenges at work.” The reliability of the scale in the present study is 0.77.

##### Sustainable tourism attitude

The sustainable attitude scale was developed by Hsu et al. (2020). It has 7 dimensions, namely, perceived social costs, perceived economic benefit, environmental sustainability, maximizing community participation, long-term planning, and community-centered economy and ensuring visitor satisfaction. We have taken an aggregate of the scale in the current study supported by CFA. The aggregate of STA shows a better fit as compared to individual items, thus we took an aggregate in our study. The reliability of the overall scale is 0.81. The CFA of one-factor model is a better fit (χ^2^ = 15, df = 0, *p* < 0.001; CFI = 0.97, NFI = 0.95, GFI = 0.93, RMSEA = 0.07) as compared to 7 factor (χ^2^ = 278, df = 58, *p* < 0.001, CFI = 0.90, NFI = 0.88, GFI = 0.86, RMSEA = 0.11).

##### Sustainable organizational performance

The scale to measure sustainable organizational performance was developed by Lee and Ha-Brookshire (2017). There are three dimensions of the scale (i) Financial Performance, (ii) Social Performance, and (iii) Environmental Performance. A total of 8 items are included in the study. A sample item is “I am aware that my company has a competitive advantage in its sales and profit growth,” “I am aware that my company has the policy to strive to be a good corporate citizen,” “I am aware that my company has the initiative to reduce, reuse, and recycle.” The overall reliability of the scale in the present study is 0.75.

We used aggregate scale of sustainable organizational performance as after conducting CFA, the 1-factor model (combined) has a better model fit (χ^2^ = 260, df = 130, *p* < 0.001; CFI = 0.96, NFI = 0.93, GFI = 0.93, RMSEA = 0.05) as compared to three factor model (χ^2^ = 609, df = 134, *p* < 0.001, CFI = 0.85, NFI = 0.81, GFI = 0.82, RMSEA = 0.12).

#### Confirmatory factor analysis

Although the current study used a time wave research design with data collected at three different time intervals from one source, a few scales have multiple dimensions. To ensure discriminant validity, we used AMOS 18 to run confirmatory factor analyses (CFAs) of variables (Table 3). We performed a pairing of CFA based on Anderson and Gerbing’s (1988) guidelines by combining a two-factor model with a one-factor model for variables that were measured at the same time and from the same source and have dimensions.

**TABLE 3:** Model fit indices for CFAs.

Model test	χ^2^	df	χ^2^/δϕ	CFI	NFI	GFI	TLI	RMR	RMSEA
**For T2**									
1 factor (ER, STA combined)	358	48	7.47	0.71	0.69	0.81	0.61	0.12	0.14
2 factor ER, STA	**91**	**43**	**2.12**	**0.96**	**0.93**	**0.95**	**0.94**	**0.06**	**0.04**
**IV and mediators**									
1 factor (CL, ER Combined)	926	193	4.82	0.71	0.66	0.75	0.65	0.19	0.11
2 factor (CL and ER)	**325**	**189**	**1.72**	**0.95**	**0.92**	**0.92**	**0.94**	**0.05**	**0.04**
**STA dimensions**									
7 factor (7 dimensions)	278	58	4.8	0.9	0.88	0.86	0.86	0.1	0.11
1 factor (7 dimensions combined)	**155**	**60**	**2.58**	**0.97**	**0.95**	**0.93**	**0.95**	**0.06**	**0.07**
**DV dimensions T3**									
3 factor (FP, SP, EP)	609	134	4.55	0.85	0.81	0.82	0.8	0.11	0.12
1 factor (FP, SP, EP Combined)	**260**	**130**	**2.01**	**0.96**	**0.93**	**0.93**	**0.95**	**0.05**	**0.05**
**IV, Mediator, DVs**									
1 factor (CL, ER, SOP Combined)	3761	726	5.12	0.52	0.47	0.5	0.46	0.31	0.36
3 factor (CL, ER, SOP)	**1165**	**711**	**1.64**	**0.95**	**0.95**	**0.93**	**0.94**	**0.06**	**0.04**
**All variables**									
1 factor (CL, ER, SOP, STA Combined)	6179	1261	4.91	0.49	0.44	0.43	0.44	0.38	0.12
4 factors (CL, ER, SOP, STA)	**1871**	**1242**	**1.57**	**0.94**	**0.9**	**0.92**	**0.93**	**0.06**	**0.04**

*N* = 306. T1 = time 1; T2 = time 2; T3 = time 3. CL, Change Leadership; ER, Employee Resilience; STA, Sustainable Tourism Attitude; SOP, Sustainable Organizational Performance. Best model fits are given in bold.

#### Results

Table 4 depicts the means, standard deviations, reliabilities, and correlations, of measures used in the current study. A one-way analysis of variance (ANOVA) was conducted to identify controls in the study. A significant difference in the department was reported, thus it was used as a control in all the analyses conducted.

**TABLE 4:** Descriptive statistics, correlation, and reliabilities.

	Mean	SD	AVE	1	2	3	4
1. CLT1	2.13	0.84	0.54	(0.80)			
2. ERT2	2.43	0.83	0.50	0.35**	(0.77)		
3. STAT2	2.55	0.87	0.52	0.40**	0.66**	(0.81)	
4. SOPT3	2.78	0.82	0.50	0.51**	0.50**	0.49**	(0.75)

**Correlation is significant at the 0.01 level (two-tailed).

*Correlation is significant at the 0.05 level (two-tailed). *N* = 306. T1 = time 1; T2 = time 2; T3 = time 3. CL, Change Leadership; ER, Employee Resilience; STA, Sustainable Tourism Attitude; SOP, Sustainable Organizational Performance.

We conducted CFA to ensure the best structural model for the study. The CFA for 1 proposed paths and 2 alternate paths was conducted. The proposed path shows the better model fit (χ^2^ = 1148, df = 747, *p* < 0.001; CFI = 0.95, NFI = 0.93, GFI = 0.92, RMSEA = 0.04) as compared to alternate paths (χ^2^ = 1367, df = 738, *p* < 0.001; CFI = 0.88, NFI = 0.81, GFI = 0.82, RMSEA = 0.06) and (χ^2^ = 1265, df = 744, *p* < 0.001; CFI = 0.89, NFI = 0.82, GFI = 0.84, RMSEA = 0.07) (Table 5).

**TABLE 5:** Comparison of alternative structural models.

	Model test	χ^2^	df	χ^2^/df	CFI	NFI	GFI	TLI	RMR	RMSEA
1	Hypothesized Model: Indirect paths from CL to SOP through ER	1148	747	1.53	0.95	0.93	0.92	0.94	0.05	0.04
2	Alternative Model 1: Direct and indirect paths ER from CL to SOP	1367	738	1.85	0.88	0.81	0.82	0.88	0.06	0.06
3	Alternative Model 2: Direct Path from CL and ER to SOP	1265	744	1.70	0.89	0.82	0.84	0.87	0.12	0.07

*N* = 306. CL, Change Leadership; ER, Employee Resilience; STA, Sustainable Tourism Attitude; SOP, Sustainable Organizational Performance. Department is controlled in all models.

The standardized direct path coefficients of the hypothesized relations show a positive relationship as predicted (Table 6). The relationship of change leadership with sustainable organizational performance (*B* = 0.28, *p* < 0.001), and employee resilience (*B* = 0.22, *p* < 0.001), was positive and significant. The direct relationship of employee resilience with sustainable organization performance was also found to be positive and significant as forecasted (*B* = 0.59, *p* < 0.001).

**TABLE 6:** Standardized direct path coefficients of the hypothesized model.

	Path	Estimate	SE
H1	CL→SOP	0.28***	0.06
H2	CL→ER	0.22***	0.06
H3	ER→SOP	0.59***	0.20

***p ≤ 0.001, **p ≤ 0.01, *p ≤ 0.05. *N* = 306. CL, Change Leadership; ER, Employee Resilience; STA, Sustainable Tourism Attitude; SOP, Sustainable Organizational Performance.

We employed the process technique devised by Preacher and Hayes (2004) to test mediation, moderation, and mediated moderation. A bootstrapping technique was deployed in SPSS micro, which was used in the current study for robust tests. To confirm moderation results, we plotted interaction plots using+1 SD and -1 SD above and below the mean. As depicted in Table 7, the mediation hypotheses (H4) were supported. The mediation results depict positive and indirect effects of change leadership on sustainable organization performance in the presence of employee resilience (Effect = 0.10, SE = 0.03, *p* < 0.001). The indirect effect using the Sobel test with an assumption of normal distribution was found to be significant and positive for sustainable organizational performance (Sobel effect = 0.10, *z* = 3.90, *p* < 0.001).

**TABLE 7:** Results of mediation hypotheses.

	Bootstrap results for direct and indirect effects (bias corrected confidence interval method)					Indirect effects using sobel			
	Paths	Effect	SE	LL 99% CI	UL 99% CI	Effect	SE	Z	*p*
H4	CL→ER→SOP	0.10	0.03	0.04	0.18	0.10	0.02	3.90	0.00

*N* = 306. Unstandardized regression coefficients are reported. CL, Change Leadership; ER, Employee Resilience; STA, Sustainable Tourism Attitude; SOP, Sustainable Organizational Performance. Bootstrap sample size = 5,000. LL, lower limit; CI, confidence interval; UL, upper limit.

Hypothesis 4 forecasts the moderating role of the sustainable tourism attitude on the relationship between employee resilience and sustainable organizational performance. Table 8 depicts that the employee resilience and sustainable tourism attitude interaction (*B* = 0.158, SE = 0.03, *p* < 0.001), was significant for sustainable organizational performance. We plotted the interaction plot to further strengthen the results.

**TABLE 8:** Results of moderated regression analyses for sustainable tourism attitude (STA) as a moderator between employee resilience (ER) and perceived sustainable organizational performance (SOP).

Predictor			B	SE	T	P
Constant			3.715	0.0389	95.517	0.000
ER			0.4775	0.0430	11.097	0.000
STA			0.0099	0.0458	0.2150	0.010
ER*STA			0.1581	0.0348	4.5468	0.000

**Conditional direct effects of X on Y**						
**IM**	**Effect**	**Boot SE**	**T**	**P**	**LLCI**	**ULCI**

STA -1 SD (–0.9907)	0.3209	0.0530	6.0592	0.000	0.2168	0.4250
STA M (0.00)	0.4775	0.0430	11.0971	0.000	0.3930	0.5620
STA +1 SD (0.9907)	0.6341	0.0572	11.0881	0.000	0.5217	0.7465

**FIGURE 2:**
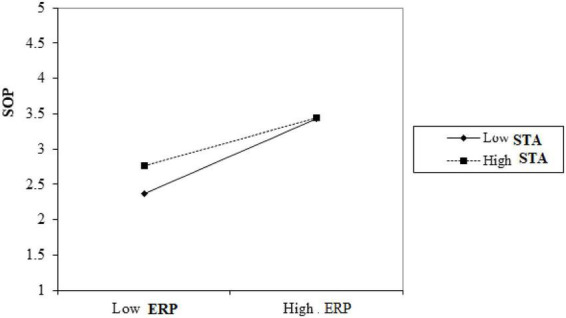
Interaction plot.

Figure 2 represents, as forecasted in Hypothesis 4, the positive relationship between employee resilience and sustainable organizational performance was positive and stronger in the presence of a high sustainable tourism attitude (β = 0.63, *t* = 11.08, *p* < 0.001), whereas it was significant and low when sustainable tourism attitude was low (β = 0.320, *t* = 6.05, *p* < 0.001). Thus, Hypothesis 4 was supported.

## Discussion

In today’s fast-growing and competitive world, business paradigms are shifting due to the uprising of sustainability-related issues, which is the new emerging contemplation in relating to human and organizational practices in every aspect. Moreover, in organizations, the vital foundation in achieving a competitive advantage to survive and compete in sustainability brings value to shifting the economic paradigm. However, the leaders are fully accountable for the efficient and effective implementation of the change process in an organization and for encouraging and motivating employees in a manner that develops the change culture successfully in an organization. Moreover, for the sustainability of an organization, there is a massive need to provide awareness and urgency among the employees for the need of adoption for change plus to develop a sense of belonging in the process of change in an organization.

In addition, the employee resilience includes difficulty and complexity at the start but they result as positive adoption of change. When the embracing of change influences employee resilience with the resource-utilizing and adaptive capacity, it leads toward developing organizational resources (De Rossi, 2013). In the past, it is found that change leadership can influence highly resilient individuals who have better coping and adaptive abilities when faced with adversity, such as work-related stress. In addition, it is found that when employees are persistently motivated and augmenting to find innovative methods and bumped into challenges by coping up with the change, they are likely to cultivate better change readiness, which encourages employee resilience (Sundblad et al., 2013).

When the organization helps and supports its employees for the development and boosting during the change process and challenging times, or the circumstances that help them to adapt to the change successfully by boosting the capability of their employees in an effective manner and employees recognize that the organization is valuing their contribution and providing them the required help, they reciprocate with efforts to increase the organizational performance (Imran and Aldaas, 2020). Employee resilience directly shapes the performance of an organization and can increase and contribute positively to organizational performance (Stajkovic et al., 2006; Luthans and Youssef, 2007; Ollier-Malaterre, 2010; Robertson et al., 2011; Blumberg et al., 2014; Scarbrough et al., 2015).

However, numerous organizations perceive employee resilience with the asset and quality traits for an organization to enhance organizational performance. Execution of resilience needs an effective leader to overcome the crisis and which is oriented to superior performance and focus on change (Malmi and Brown, 2008). Moreover, an organizational culture by proactively implementing change leadership can create employee resilience, which leads toward the sustainability, enhancement, and success of the organization (Alesi, 2008). The finding reveals that organizational resilience is positively and significantly associated with organizational performance (Malik and Garg, 2017). Change leadership and employee resilience in the organization play an important role in organizational performance to meet all the challenges and issues that arise during tough situations.

### Theoretical implications

This research study provides in advance, understanding during the critical times of COVID-19 pandemic crisis happening around the globe, how the tourism industry has suffered, as well as the rate of poverty increased in terms of lack of business sustainability in the tourism industry due to restrictions on the tourist spots and travel has suffered and ruined the tourism contribution in an economy. However, in these times an organization can achieve a competitive advantage by implementing a change paradigm successfully by creating employee resilience by maintaining a healthy work environment, which leads to a sustainable organizational performance. The current research extends the body of literature by providing enriched conceptualization of how leaders can achieve success by increasing sustainable organizational performance and specifically in the tourism sector.

### Managerial implications

The study shed light on the multiple aspects, such as change which has been the only constant in the quests for sustainable tourism presenting the industry with economic, environmental, as well as social issues that require substantial changes in traditions and attitudes of stakeholders of the said industry. Issues such as severe climatic changes, poverty, resource shortages, unstable politics, and globalization have been grabbing the attention of researchers and practitioners in light of the United Nation’s Sustainable development goals (Caffaro et al., 2019). Moreover, an organizational culture proactively implementing change leadership can create employee resilience, which leads toward the sustainability, enhancement, and success of the organization. The finding reveals that organizational resilience is positively and significantly associated with organizational performance. The current study in this context serves as a source to open policy making and further investigations to explore the tourism industry to curb issues such as climatic changes and poverty in a progressing economy such as Pakistan.

### Limitations and future directions

This research study contributes in numerous aspects such as the employee’s perception of change leadership and its impact on their resilience toward the sustainable organization performance of companies’ operationalizing in the sector with moderating impact of sustainable tourism attitude in times of crises. However, various organizations perceive employee resilience as the asset and a quality trait for organizations to enhance organizational performance. Execution of resilience needs an effective leader to overcome the crisis and which is oriented to superior performance and focus on change. In addition, where there are various research contributions, there are a few limitations that can be addressed in future research studies, such as this research study is based on cross-sectional data where in future the research based on longitudinal data can also be conducted. On the other hand, this research study is limited to the specific context such as the tourism industry, but it can also be done in the other sectors/industries and contexts such as telecommunication sector, textile, and construction. Future research can also be conducted by adding the employee turnover intention in the relationship between change leadership and sustainable organizational performance.

## Conclusion

As the study aimed to look at the role of leadership as a source of growth in eradicating poverty in the Pakistani economy by focusing on the tourism industry, the researchers have found evidence in support of the propositions of the study. There have been many studies in the past that have looked at the role of multiple leadership styles and how they can improve the productivity of the employees in different sectors. The current study had a unique factor of applying a mixed method approach to first identify the problems in the selected industry and then conjugate empirical gaps and survey results to confirm the claims. Tourism is the focus of the Pakistani government at the moment, and this adds to the contextual value of the study. The findings have provided in-depth insights into the policy makers in the industry to understand how sustainability in the tourism industry can be encouraged by promoting a change attitude in leaders and a resilient attitude in the workers of the industry. The study further opens avenues for researchers to investigate boosters for the industry as tourism plays an important role not just in growing economies but also in healthy lifestyles contributing to the mental health of individuals.

## Data availability statement

The raw data supporting the conclusions of this article will be made available by the authors, without undue reservation.

## Ethics statement

Ethical review and approval was not required for the study on human participants in accordance with the local legislation and institutional requirements. Written informed consent from the patients/participants or patients/participants legal guardian/next of kin was not required to participate in this study in accordance with the national legislation and the institutional requirements.

## Author contributions

FB conceived the idea of the study. ZT and AA contributed to the data collection, analysis, and writing up respectively under the leadership of FB. All authors played a very critical role in the completion of the study in terms of data collection, analysis, and reporting.
